# Atherosclerosis of cavernosal arteries as a cause of erectile dysfunction in an adult: our findings on triplex doppler sonography

**DOI:** 10.4314/gmj.v55i1.12

**Published:** 2021-03

**Authors:** Joshua O Aiyekomogbon, Ukamaka D Itanyi, Terkaa Atim, Sadiq Abu

**Affiliations:** 1 Department of Radiology University of Abuja Teaching Hospital, Abuja, Nigeria; 2 Department of Radiology, Federal Medical Centre, Abuja, Nigeria; 3 Department of Surgery, University of Abuja Teaching Hospital, Abuja, Nigeria

**Keywords:** Atherosclerosis, arteriogenic ED, Prostaglandin E_1_, Doppler, ultrasound

## Abstract

A sixty year old man presented to our health facility with a year history of severe erectile dysfunction (International Index of Erectile Function Score of 5 (IIEF-5)). He was also a known hypertensive and currently being managed for hypertensive heart disease by the Cardiologist. Colour Doppler interrogation of the Cavernosal Arteries showed multiple areas of narrowing in both arteries, giving beaded appearance. The peak systolic velocities of the arteries were less than 25cm/s, and there was persistent diastolic flow in the entire spectral recordings, prompting the diagnosis of arteriogenic erectile dysfunction (ED) secondary to atherosclerosis of the cavernosal arteries. He had medical treatment with PGE5-I, and intracavernosal injection of prostaglandin E_1_ (Caverjet), but all to no avail. He was counselled for penile implant and he is favourably disposed to that but largely being limited by funds.

**Funding:**

None Declared

## Introduction

Erectile dysfunction is a major problem in men irrespective of age, race or social class, and it is defined as consistent inability to achieve and maintain erection of sufficient rigidity for satisfactory sexual performance. It is particularly common in men above the age of 50 years, and its causes could be organic, psychogenic or both. Vasogenic causes are the commonest and these could be arteriogenic, venogenic or both and treatment options are largely dependent on the aetiologic categorization.

With aging, there are co-morbidities that could be associated with ED, and most of these express themselves as arteriogenic or venogenic dysfunction.

We present a case of a sixty year old man with erectile dysfunction whose symptoms persisted despite prolonged medical treatment until he benefitted from triplex Colour Doppler examination of the penis where diagnosis of arteriogenic ED secondary to cavernosal arteries atherosclerosis was made. This brings to the fore the need for colour Doppler study of the cavernosal arteries before instituting any form of management as treatment options are cause-specific.

## Case Report

A 60-year- old married man with one year complaints of severe erectile dysfunction with International Index of erectile function score of 5 (IIEF-5). There was no loss of libido or nocturnal erections. He neither smoke cigarette nor ingest alcohol and there was no past history of pelvic trauma. He was a known hypertensive for over ten years and currently being managed for hypertensive heart disease with spirinolactone, idapamide and telmisatan. He had open prostatectomy for benign prostatic enlargement eight months prior to presentation. He was originally married to two wives but had to divorce the second wife as a result of the poor erection status. His physical examination findings were unremarkable. He had mild cardiomegaly with the apex beat at sixth left intercostal space anterior axillary line. His pulse rate was 90/min while the blood pressure was 140/90mmHg. The heart sounds were normal S_1_ and S_2_. No added sounds and no murmur were heard. The **remaining** systems were essentially normal.

He had normal renal function test, lipid profile, and fasting blood sugar. Electrocardiography revealed first degree heart block, left bundle branch block (LBBB), left atrial enlargement (LAE) with features of hypertensive heart disease and reduced ejection fraction. Right atrial enlargement, left ventricular hypertrophy, impaired left ventricular and right ventricular systolic function, and EF of 32% were also noted on Echocardiography. He was treated with phosphodiesterase type 5 inhibitors (PDE5-I) (sidanafil and tadalafil) for one month but no improvement of erectile function was noted and this necessitated referral to Radiologist for penile Doppler study.

**Figure 1:**
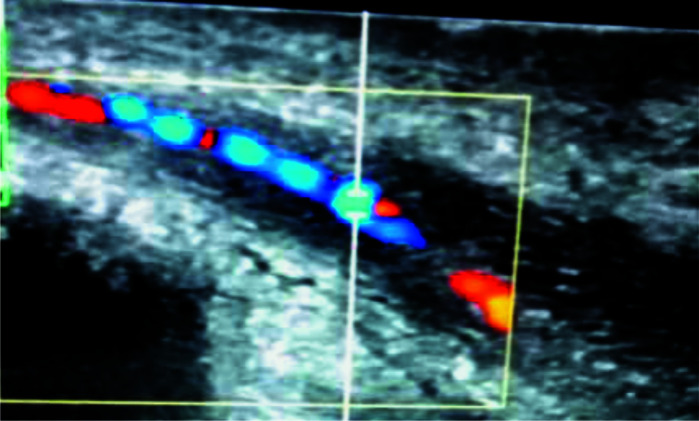
Left CA colour Doppler showing multiple areas of narrowing and dilatations, giving beaded appearance

**Figure 2:**
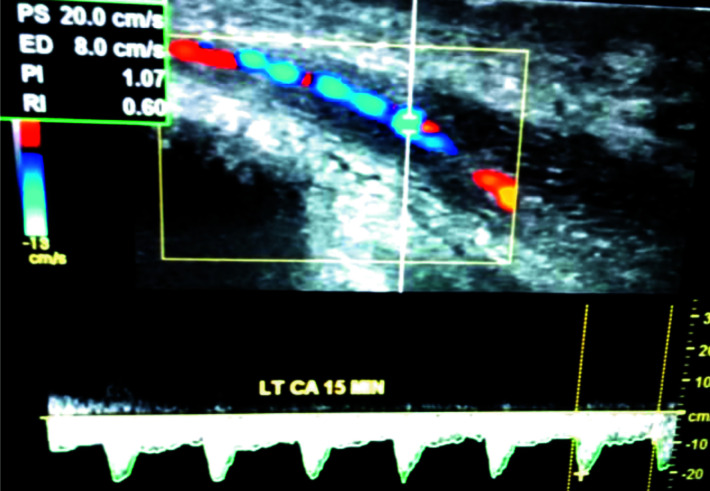
Left CA triplex Doppler waveform at 15 minutes showing multiple areas of narrowing and dilatations, giving a beaded appearance. Note the peak systolic velocity of 20cm/s, and end diastolic velocity of 8cm/s

**Figures 3a and 3b:**
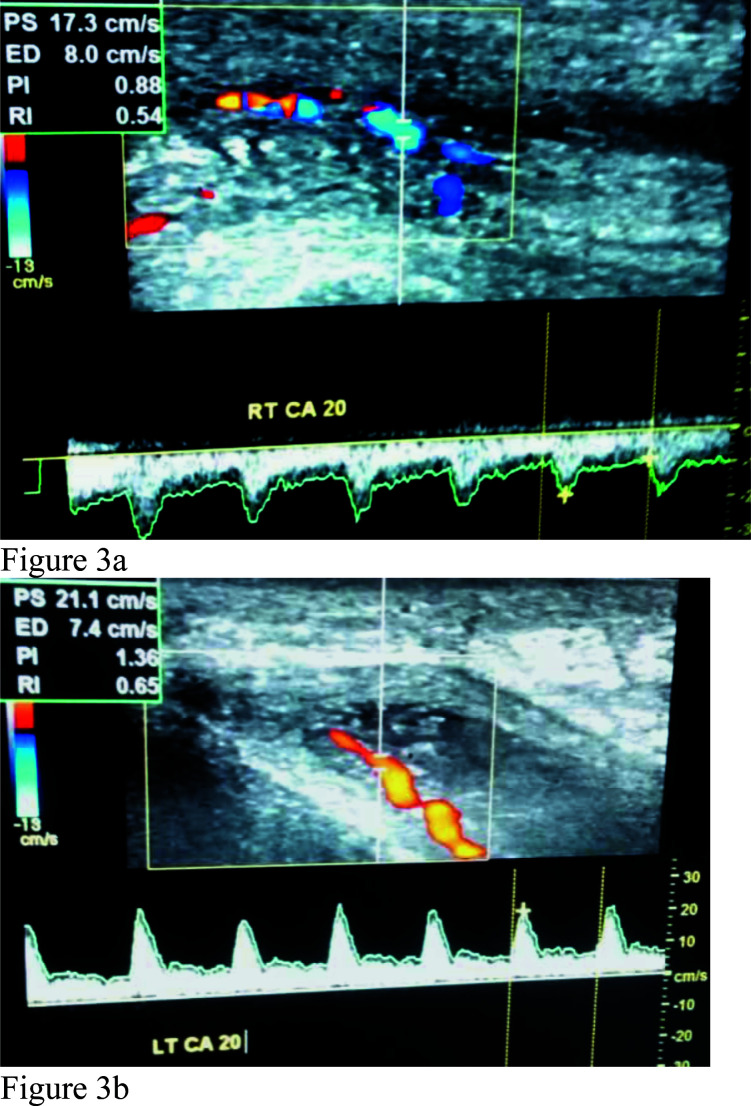
Triplex Doppler waveforms of the right and left cavernosal arteries at 20 minutes showing multiple areas of narrowing and dilatations, giving beaded appearances. Note the peak systolic velocities of 17.3cm/s and 21.1cm/s on the right and left side respectively.

Doppler assessment of the cavernosal arteries following intracavernosal injection of 20µg of prostaglandin E_1_-PGE_1_ (Figures 1–3) revealed multiple areas of narrowing and post-stenotic dilatations in both cavernosal arteries, giving beaded appearance. The peak systolic velocities of the arteries also were less than 25cm/s, and there was persistent diastolic flow in the cavernosal arteries in the entire spectral recordings with end diastolic velocity (EDV) greater than 5cm/s at all levels. These features prompted the diagnosis of arteriogenic erectile dysfunction secondary to atherosclerosis of the cavernosal arteries.

He was placed on Trimix but all to no avail, and he later had intracavernosal injection of 10 to 20µg of Prostaglandin E_1_ (PGE_1_) on three occasions but had poor response. He is now being counselled for penile implant which he's favourably disposed to but largely being limited by funds.

Erectile dysfunction in this patient could be multi-factorial. However, the characteristic penile Doppler features of cavernosal atherosclerotic changes with a normal fasting lipid profile is worthy of note.

## Discussion

Vasculogenic erectile dysfunction is the most common organic causes of erectile dysfunction in men.^1^ Obstruction of blood flow to the corporal bodies by atherosclerotic lesions is responsible for ED and this can be due to endothelial damage or penile arterial injury.^1^,^2^ The index case had atherosclerotic changes of the cavernosal arteries resulting in multiple areas of narrowing, giving a classical beaded appearance. This may explain his poor response to medical treatment.

Diagnosis of arteriogenic erectile dysfunction in men aged 50 years and above should prompt the attending physician to evaluate the patient for the presence of vascular disease outside the penile bed.^3^–^5^ A strong association is established between arteriogenic erectile dysfunction and high prevalence of clinically apparent atherosclerosis.^5^ A study of 45 men by Greenstein *et al^5^* established a significant correlation between coronary arteries occlusion on angiography and presence of arteriogenic ED hence, holistic evaluation of this category of patients is strongly recommended. Arteriogenic ED is therefore a ‘blessing in disguise’ as proactive measures by the attending physician can go a long way to avert a more devastating sequalae of infarctive stroke and cardiac arrest which are common causes of death in our environment.^5^ In view of this, coupled with the fact that this patient is a known hypertensive, he is also currently been evaluated by the Cardiologist and so far he is stable.

Altered Nitric Oxide (NO) synthesis is a peculiar problem as men ages, and the index case was a 60 year old man and it could therefore be one of the factors responsible for arteriogenic ED in him. Haas *et al^6^* showed an upregulation of endothelial Nitric Oxide synthase (eNOs) in the corporal smooth muscle cells and the endothelial of the ageing rabbits. This was corroborated by the Massachusetts Male Ageing human studies of Feldman *et al^7^* and *Johannes et al^3^* which showed that increasing age was the most strongly associated factor responsible for ED. Other factors implicated in these studies were Diabetes Mellitus, Low HDL (high density lipoprotein), Systemic Hypertension and lower educational status. This index case was 60 years and a known hypertensive. These factors combined may be responsible for the arteriogenic ED observed in him. The relationship of systemic hypertension with ED is largely due to vascular endothelial damage, use of centrally acting antihypertensive drugs, lower serum testosterone levels, and testosterone is known to stimulate cavernosal Nitric Oxide (NO) production. NO helps in the relaxation of the cavernous sinusoids during normal erection and its absence leads to poor distension of the sinusoids, absent turgidity of the penis and ultimately, poor erection.^6^

Also, a study of Toblli *et al^8^* showed significant increase in cavernosal and vascular smooth muscle proliferation and cavernosal fibrosis in hypertensive model than controls. These findings are possibilities in the index case considering his hypertensive status. In studies conducted by Aiyekomogbon *et al^9^,^10^* among Nigerians, it was discovered that cavernosal arterial peak systolic velocity decreases with age and arteriogenic ED was commoner in the patients above 40 years of age therefore;arteriogenic ED in this man is not surprising considering his age.

Chronic cigarette smoking is another aetiologic factor for arteriogenic erectile dysfunction because of the effect of smoke on the vascular endothelium and peripheral nerves but the index case has never smoked, and his lipid profile is essentially preserved. 11–13 It is worthy of note that the index case had a low left ventricular ejection fraction (LVEF) of 32%, and Baumhakel*et al 14* observed in their work that arterial endothelial dysfunction (cavernosal arteries inclusive), is common in patients with low LVEF which ultimately results in decreased erectile function. In view of this observation, the low ejection fraction in this patient could be one of the precursors for the development of the observed atherosclerosis of the cavernosal arteries.

The percentage stenosis of the index case based on colour Doppler interrogation is greater than 70%, and, Azadzoi& Saenz^12^ observed in their study that arteriogenic ED occurs when more than 50% of the lumen of internal pudendal, common penile or cavernosal arteries is occluded.^12^ This may account for the severity of the erectile dysfunction and his poor response to the medical treatment. Angiography also has a role to play in establishing these atherosclerotic changes particularly when proximal vessels such as internal pudendal and internal iliac arteries are involved.^15^ Atherosclerotic changes in these set of vessels may not be amenable to sonographic diagnosis own to respiratory movements and effects of the overlying bowel gas shadows which may affect penetration of sound waves. This patient may require this in the nearest future particularly at the time of interventional management as angiography is both diagnostic and therapeutic. The invasiveness of angiographic technique and use of ionizing radiation are the known drawbacks but these are not sufficiently strong enough to negate its relevance in this case scenario. In venous leak also, high resolution images of the venous drainage using a 3D-Computed Tomography Cavernosography has a vital role to play in the localization of the actual veins that are responsible for the leakage.^16^ In the case under consideration however, this may not be applicable as cavernosal veno-occlusive disease (CVOD) has not been established as a cause of erectile dysfunction in the patient.

No single treatment protocol is applicable to all cases of arteriogenic erectile dysfunction.^15^ Treatment is decided on a case-by-case basis, which is usually medical, using oral phosphodiesterase type 5 inhibitors (PGE5-I) such as sildenafil, tadalafil, or vardenafil. Option of intracavernosal injection of prostaglandin E_1_ is the second line of care and he benefitted from both options but all to no avail. Penile implant is another option which our patient is favourably disposed to but he is been largely limited by funds. Use of penile pump has been advocated in some cases.^15^ Radiology and surgical interventional management with arterial stenting, balloon dilatation of the stenosed segments of the cavernosal arteries, and penile arterial bypass are also possibilities but expertise and requisite equipments are not available in our setting. If that is desired and affordable by the patient he will be refereed appropriately.

## Conclusion

The relevance of triplex Doppler sonography of the penis in the diagnosis of atherosclerosis of cavernosal arteries which resulted in arteriogenic erectile dysfunction is brought to limelight in this report. It is therefore imperative that patients with ED benefit from this safe, non-invasive and non-ionizing imaging protocol before initiating treatment as that will go a long way to prevent treatment failure and unnecessary waste of monetary resources on blind management of such cases.
